# Nivolumab Effective for Gastric and Lung Cancers but Not for Multiple Myeloma in a Multiple Primary Cancer Patient

**DOI:** 10.1155/2021/9965371

**Published:** 2021-08-26

**Authors:** Tsukasa Yasuda, Junji Hiraga, Michihiko Narita, Yoshimasa Tanikawa, Tomoyuki Tsuzuki

**Affiliations:** ^1^Department of Gastroenterology, Toyota Kosei Hospital, Toyota, Japan; ^2^Department of Hematology, Toyota Kosei Hospital, Toyota, Japan; ^3^Department of Pathology, Toyota Kosei Hospital, Toyota, Japan; ^4^Department of Respiratory Medicine, Toyota Kosei Hospital, Toyota, Japan

## Abstract

The case of a 76-year-old man with multiple primary cancers that were treated with nivolumab is presented. Six years earlier, he was diagnosed with multiple myeloma (MM) and was treated with several chemotherapies. He was also diagnosed with gastric cancer with liver metastasis and primary lung cancer by upper gastrointestinal endoscopy and computed tomography (CT). Nivolumab treatment was given as third-line therapy, and it was effective for gastric and lung cancers. But MM worsened, and the patient died. There is no standard treatment for multiple primary cancers, and the development of effective treatments for multiple primary cancers is important.

## 1. Introduction

Multiple myeloma (MM) is a hematological malignancy for which survival has been prolonged through the development of new agents such as proteasome inhibitors, immunomodulators, and antibody drugs [1–3]. The median survival of patients with MM has increased from 3 to 8 years in the last 15 years. However, second primary cancers have created problems with the long-term survival of patients with MM [4]. It has been reported that the risk of developing solid tumors remains unchanged, but the risk of developing hematological malignancies is significantly higher, especially in patients treated with immunomodulators such as lenalidomide [5–7]. In addition, the prognosis of patients with second primary cancers has been reported to be worse than that of patients without them [8, 9].

Nivolumab is classified as a checkpoint inhibitor that is a monoclonal antibody drug against programmed cell death-1 (PD-1). The indications for PD-1 antibody drugs have been expanded to various cancers, and it has become available from the second line for unresectable advanced or recurrent gastric cancer and the first line for unresectable advanced or recurrent non-small cell lung cancer [10, 11]. In contrast, unfortunately, pembrolizumab, a similar PD-1 antibody, was found not effective for MM in combination with lenalidomide [12], so the PD-1 antibody drugs are not approved for MM in the United States and Japan.

A rare case, in which a patient who had been treated with new agents for MM developed multiple primary cancers (gastric cancer and lung cancer) and was treated with nivolumab, which was effective for gastric and lung cancers, but not for MM, is presented.

## 2. Case Report

**Figure 1:**
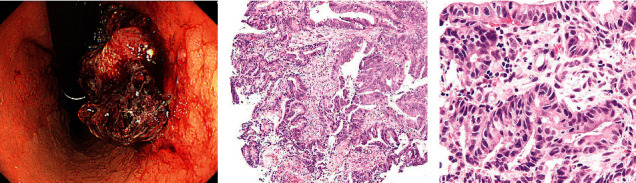
(a) Upper gastrointestinal endoscopy findings. A type 1 tumor with a diameter of 30 mm is found on the posterior wall of the gastric body. The tumor is easily bleeding. (b) Histopathologic findings of the biopsy specimens of gastric cancer. Findings of well-differentiated adenocarcinoma. Hematoxylin and eosin staining, ×100. (c) Hematoxylin and eosin staining, ×400.

**Figure 2:**
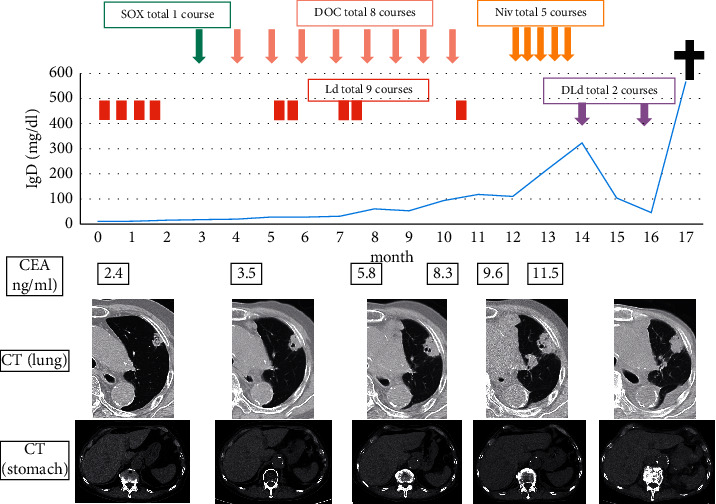
Clinical courses of the patient after gastric and lung cancers are diagnosed. The solid line indicates serum IgD level. Computed tomography (CT) shows the lung nodules and stomach. At the time of appearance, a pulmonary nodule with a diameter of 20 mm is found in the upper lobe of the left lung. The tumor grew over time, and a new intrapulmonary lesion appears after DOC treatment, but after nivolumab treatment, the new lesion almost disappeared and the primary lesion reduced. The gastric cancer could not be identified by CT at the start of treatment and had not changed since then. But 16 months after, gastric cancer increased. SOX: tegafur, gimeracil, oteracil potassium (TS-1), and oxaliplatin; DOC: docetaxel; Niv: nivolumab; Ld: lenalidomide and dexamethasone; DLd: daratumumab, lenalidomide, and dexamethasone, CEA: carcinoembryonic antigen.

A 76-year-old man noticed melena and had a medical examination. He was found to have renal dysfunction 6 years earlier, and further evaluations showed serum albumin, 2.2 g/dL; urinary protein, 4+; urinary Bence Jones protein- (BJP-) *λ*-type M protein positivity; decreased serum IgG, IgA, and IgM levels; and an increased serum IgD (516 mg/dL) level. In addition, bone marrow aspiration showed clonal proliferation of plasma cells, so he was diagnosed with MM (IgD-*λ* type, BJP-*λ* type, International Staging System III). He was treated with 7 courses of bortezomib dexamethasone (BD) therapy, but due to recurrence, his therapy was changed to lenalidomide dexamethasone (Ld), and he was undergoing his 36th course. On upper gastrointestinal endoscopy, a type 1 tumor with a diameter of 30 mm was seen at the posterior wall of the corpus of the stomach, and the histopathological diagnosis of the tumor was tubular adenocarcinoma (Figures 1(a)–1(c)). And, the HER2 status of gastric cancer was negative, and MSI status of that was not examined. In addition, computed tomography (CT) showed liver metastasis of gastric cancer, so the gastric cancer was diagnosed as clinical stage IV (T3N2M1). Furthermore, CT showed a pulmonary nodule with a diameter of 20 mm in the upper lobe of the left lung, and the diagnosis of the nodule was primary lung cancer clinical stage IA2 (T1bN0M0) based on the imaging findings (Figure 2). Transbronchial lung biopsy could not be performed because the lesion of the lung cancer was a very difficult place for the examination, and the gastric cancer had advanced and a poor prognosis. So, immediate chemotherapy was needed. In conclusion, this patient was diagnosed as having primary gastric cancer and lung cancer that developed during the treatment of MM. The laboratory data at the time of diagnosis are shown in Table 1.

For this case of multiple primary cancers, the treatment plan was considered by the cancer board of our institute, and the treatment of the gastric cancer, which had the worst prognosis, was prioritized. First, one course of tegafur, gimeracil, oteracil potassium (TS-1), and oxaliplatin (SOX) was administered, but it was discontinued due to gastrointestinal symptoms and changed to docetaxel (DOC). He had a history of unstable angina and paroxysmal atrial fibrillation and had been treated for percutaneous coronary intervention two years ago. To avoid the onset of thrombosis, he was treated with monotherapy of docetaxel for second line. Since the serum IgD level also tended to increase gradually during the previous treatment, the treatment plan was re-examined, and the treatment for MM was resumed after the administration of a second course of DOC. Then, DOC and Ld treatment were administered alternately. However, due to progression of the lung cancer, the treatment was changed from DOC to nivolumab the following year. After the start of nivolumab, the lung cancer showed partial response, and the gastric cancer showed stable disease, but the MM gradually worsened. Although the serum IgD level decreased due to the addition of daratumumab, lenalidomide, and dexamethasone (DLd), the patient was unable to continue chemotherapy because of loss of appetite, which was thought to be an exacerbation of gastric cancer. The serum IgD level and carcinoembryonic antigen (CEA) level were increased. The patient died 17 months after the multiple primary cancers were diagnosed (Figure 2).

## 3. Discussion

A rare case, in which nivolumab treatment was administered for primary gastric cancer and primary lung cancer that developed during treatment for MM, and the gastric cancer and lung cancer responded, but the MM did not, was reported. Patients with second primary cancers that develop after treatment for MM have been reported to have a 2- to 6-fold increased risk of death compared to those without second primary cancers [8, 9]. In particular, patients treated with lenalidomide have a high incidence of secondary carcinogenesis, which is estimated to be 5–8% [5–7]. In addition, older age is also considered to be a significant risk factor for the development of second primary cancers [13]. According to a Swedish report, 1,547 of 26,627 patients with MM developed second primary cancers, but the rate of gastrointestinal cancers was low (364 patients, 1.4%) and that of respiratory cancers was also low (68 patients, 0.25%). A study of 2,732 patients with MM treated with lenalidomide found no gastric cancers, 5 (0.18%) lung cancers, and only 3 multiple primary solid cancer cases [13]. It is considered that cases such as the present one in which gastric cancer and lung cancer developed as second primary cancers are extremely rare. The present patient was an elderly patient who had been receiving long-term lenalidomide and dexamethasone treatments with relapse of MM after proteasome inhibitor treatment. Because the risk of death from MM is much higher than the risk of death from second primary cancers, lenalidomide treatment should be given aggressively for MM, but it is necessary to always keep in mind the onset of second primary cancers, especially in elderly patients receiving long-term lenalidomide treatment.

Nivolumab is classified as a checkpoint inhibitor that is a monoclonal antibody drug against PD-1. This drug can be used in first-line, second-line, and third-line treatments for unresectable gastric cancer and lung cancer [10, 11]. In the present case, there was no pathological proof of lung cancer, but the pulmonary nodule was likely to be primary lung cancer based on the CT findings. Gastric cancer and lung cancer were evaluated as stable disease and partial remission, respectively, based on clinical and imaging findings. However, nivolumab was not effective for MM, so daratumumab was also used, but it was also ineffective, and the patient died. Pembrolizumab, a PD-1 antibody similar to nivolumab, also failed to show efficacy against MM [12], which led to the discontinuation of clinical trials with nivolumab. Therefore, PD-1 antibody drugs are not indicated for MM. PD-1 antibody drugs have been reported to be effective for MM in some cases [14], but not in the present case. Other therapeutic approaches may be needed for MM, but in the present case, treatment for MM alone was associated with a high risk of exacerbation of other multiple cancers, so further approaches may have been difficult.

With the development of treatment for MM, the diagnosis and treatment of multiple primary cancers become very important issues. The present case was treated with nivolumab, but it was ineffective for MM. With the accumulation of more such multiple primary cancers cases, more effective treatments might be developed.

## Figures and Tables

**Table 1:** Laboratory data at the time of diagnosis of gastric cancer and lung cancer.

		Units	Reference range
*Hematology*			
WBC	7,300	*μ*L	4,000–9,000
Neut	52	%	38.0–71.9
Eos	2.0	%	0.2–6.8
Ba	0.0	%	0.0–1.0
Mono	8.0	%	2.3–7.7
Lymph	38	%	26.0–46.6
RBC	329	×10^4^/*μ*L	410–560
Hb	11.1	g/dL	13.5–17.5
Ht	33.5	%	40.0–53.0
MCV	89.6	fL	80.0–100.0
Platelets	20.1	×10^4^/*μ*L	14.0–40.0

*Biochemistry*			
TP	5.3	g/dL	6.7–8.3
Alb	3.6	g/dL	4.0–5.0
T-bil	0.4	mg/dL	0.3–1.2
AST	16	U/L	13–33
ALT	27	U/L	6–30
LDH	243	U/L	119–229
*γ*-GTP	106	U/L	10–47
BUN	14.9	mg/dL	8.0–22.0
Cre	0.86	mg/dL	0.60–1.10
Na	142	mmol/L	138–146
K	3.7	mmol/L	3.6–4.9
Cl	110	mmol/L	99–109
Ca	8.9	mg/dL	8.7–10.3
P	3.1	mg/dL	2.5–4.7
IgG	453	mg/dL	870–1700
IgA	33	mg/dL	110–410
IgM	21	mg/dL	35–220
IgD	10.5	mg/dL	<9.0

*Tumor markers*			
CEA	2.4	ng/mL	<5.0
CYFRA	1.4	ng/mL	<3.5
Pro-GRP	66.6	pg/mL	<81.0

*Urinalysis*			
Protein	0.22	g/gCr	<0.15
RBC	(—)		
WBC	(—)		

WBC: white blood cells; Neut: neutrophils; Eos: eosinophils; Ba: basophils; Mono: monocytes; Lymph: lymphocytes; RBC: red blood cells; Hb: hemoglobin; Ht: hematocrit; MCV: mean corpuscular volume; TP: total protein; Alb: albumin; T-Bil: total bilirubin; AST: aspartate aminotransferase; ALT: alanine aminotransferase; LDH: lactate dehydrogenase; *γ*-GTP: gamma-glutamyl transpeptidase; BUN: blood urea nitrogen; Cre: creatinine; Na: sodium; K: potassium; Cl: chloride; Ca: calcium; P: phosphorus; IgG: immunoglobulin G; IgA: immunoglobulin A; IgM: immunoglobulin M; IgD: immunoglobulin D; CEA: carcinoembryonic antigen; CYFRA: cytokeratin-19; Pro-GRP: progastrin releasing peptide.

## Data Availability

The figure data used to support the findings of this study are included within the article.
